# Multiscale Analyses of Surface Failure Mechanism of Single-Crystal Silicon during Micro-Milling Process

**DOI:** 10.3390/ma10121424

**Published:** 2017-12-13

**Authors:** Jinxuan Bai, Qingshun Bai, Zhen Tong

**Affiliations:** 1 School of Mechanical and Electrical Engineering, Harbin Institute of Technology, Harbin 150001, China; jinxuanbai@hit.edu.cn; 2 Centre for Precision Technologies, University of Huddersfield, Huddersfield HD1 3DH, UK; Z.Tong@hud.ac.uk

**Keywords:** monocrystalline silicon, brittle material, surface failure behavior, discrete dislocation plasticity, crack

## Abstract

This article presents an experimental investigation on ductile-mode micro-milling of monocrystalline silicon using polycrystalline diamond (PCD) end mills. Experimental results indicate that the irregular fluctuation of cutting force always induces machined surface failure, even in ductile mode. The internal mechanism has not been investigated so far. The multiscale discrete dislocation plasticity framework was used to predict the dislocation structure and strain evolution under the discontinuous cutting process. The results showed that a mass of dislocations can be generated and affected in silicon crystal. The dislocation density, multiplication rate, and microstructure strongly depend on the milling conditions. In particular, transient impulse load can provide a great potential for material strength by forming dislocations entanglement structure. The continuous irregular cutting process can induce persistent slip bands (PSBs) in substrate surface, which would result in stress concentration and inhomogeneous deformation within grains.

## 1. Introduction

Recently, silicon-based micro-electro-mechanical systems (MEMS) and nano-electro-mechanical system (NEMS) have been widely used in spaceflight, energy, life sciences, and medical fields due to their reliable and reproducible mechanical and electrical properties [1,2]. Ultra-precision machining technology is one of the most advanced methods for processing monocrystalline silicon devices [3,4]. Based on the principle of brittle–ductile transformation, Fang et al. [5] investigated the removal mechanism of brittle materials and found that plastic deformation was predominant when the undeformed chip thickness achieved the critical criteria of ductile removal. Similarly, Rusnaldy et al. [6,7] conducted ductile milling experiments to fabricate desired three-dimensional free-surface on silicon-based devices. Although plenty of studies have indicated that the ratio of thrust force to feed force determined the removal performance of silicon [8,9], present experiments show that the ductile processing model puts forward strict requirements for the shape-structure and amplitude of cutting force as well. Wang et al. [10] suggested that the monitoring of cutting force had great potential for high value-added cutting purposes. Actually, once a stable machining process is broken by the inappropriate cutting parameters and irregular micro-vibration, the cutting force can form an approximate shock wave in the vertical axial, which may result in the failure of ductile-mode cutting and degrade the integrity of the machined surface [11]. However, the internal mechanism of this phenomena is rarely discussed.

Monocrystalline silicon is brittle, but it has strong dislocation activity in exceptional circumstance [12,13]. Cai et al. [14] studied the dislocation velocity of monocrystalline silicon by relating mechanistic treatment to activation energy in the experimental temperature and stress. Rabier et al. [15] indicated that the plastic deformation of silicon crystal arose from a large number of dislocations with 1/2*a* Burgers on {111} slip system. Although some studies have suggested that the plasticity of monocrystalline silicon is closely associated with the phase transformation behavior, recent evidence indicated that silicon surface can lead to preferential dislocation nucleation and suppress phase transition [16]. Revealing the mechanism of dislocation motion in silicon crystal is restricted by two issues at present: (1) the severe condition for dislocation activity; and (2) the special pressure for creating and transporting the discrete dislocation [17]. Various attempts have been adopted to quantitatively reveal the evolution rule of microstructure for hard and brittle materials [18,19]. Specifically, Cheng et al. [20] carried out computational simulation to demonstrate the dislocation movement in silicon crystal. Although lots of numerical simulation methods have been conducted to assert the plastic deformation of silicon substrate, it is difficult to uncover the essences of dislocation evolution and crack initiation due to the restriction of simulation scale [21,22]. Moreover, most of the existing studies focused on local defect nucleation behavior under monotonous loading. The damages in microstructure evolution under complex cutting forces has not received enough attention.

A newly developed mesoscale analogue technique named discrete dislocation plasticity (DDP) can provide a better scalability to carry out more complicated problems, relative to the molecular dynamics (MD) method, with greater temporal scale and spatial scale [23,24]. In the DDP framework, dislocations are modeled as line defects and corresponding complementary boundaries can be obtained from finite element-based solutions. Long-range interactions among dislocations are directly solved by the elasticity model, while short-range phenomena including dislocation nucleation, motion, junction, accumulation, recovery, and annihilation are incorporated by constitutive laws [25,26]. Shishvan et al. [27] proved that a simpler two-dimensional DDP framework was more successful than the three-dimensional method in revealing the microstructure evolution and plastic deformation. Liao et al. [28] adopted discrete dislocation dynamics to investigate the pile-up effect caused by precipitates under shock wave. Results showed that the density, size, and space distribution of nano-precipitates significantly affected the pinning behavior. In particular, since the poor dislocation activity of monocrystalline silicon that results in the characteristic time of deformation is much smaller than sound speed, the DDP method is deemed as one of the most effective ways to capture the evolution rule of dislocation [29].

In this work, milling experiments and a multiscale DDP framework were carried out to study the brittle–ductile transition behavior and failure mechanism of monocrystalline silicon. Furthermore, dislocation activity, i.e., multiplication and propagation, under transient impact loading, and the continuous irregular milling process were considered in the DDP simulation model. The evolution process of mechanical property and dislocation configuration were analyzed to understand the residual surface damage on the machined surface.

## 2. Experiment Set-Up and Results

**Figure 1:**
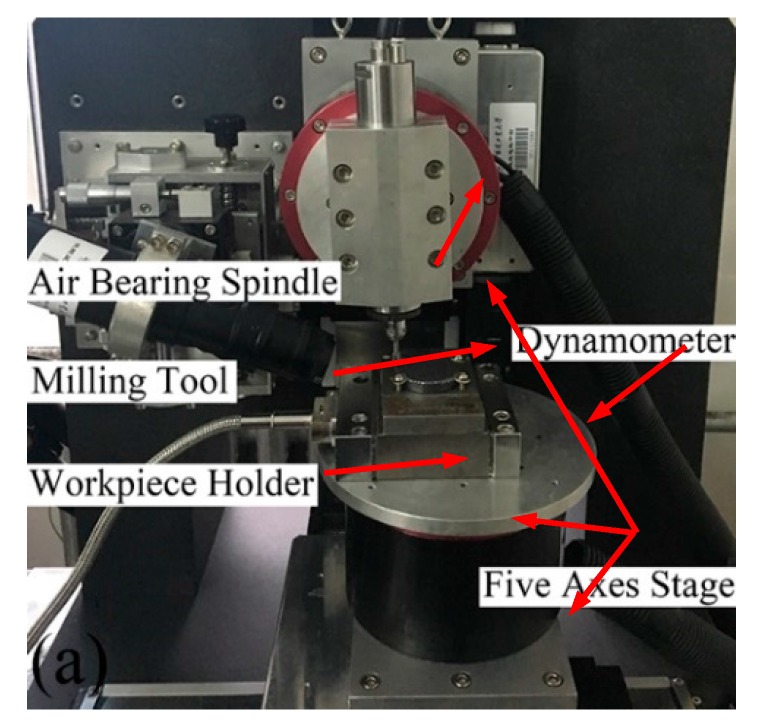
The experimental set-up for milling monocrystalline silicon.

In order to obtain the failure loading conditions for the simulation, a series of micro-machining tests for monocrystalline silicon were conducted on a self-developed five-axis milling numerical control machine tool, as shown in Figure 1. The external dimensions of machine tool were 700 mm × 580 mm × 500 mm. The air-bearing spindle could impart a maximum velocity of up to 8 × 10^4^ rpm, and the runout is less than 1 μm. The straightness positional precision could reach ±0.35 μm/10 mm. Furthermore, identical tool structural parameters of brand-new polycrystalline diamond (PCD) micro-end mills were used in machining experiments. The PCD mills have two flutes, nominal mill diameter of 5 mm, edge radius of 40 μm, nominal rank angle of −2°, handle length of 25 mm, and handle diameter of 3 mm. The (1 1 1) monocrystalline silicon workpiece was bonded to a ground metal plate. The cutting parameters are listed in Table 1. Each set of experimental conditions was repeated once to reduce the machining errors and separate the vibration effects from the interactions between machine and cutting tool. For each experiment, a 20-mm-long and 5-mm-wide micro-slot was milled and the direction of feed was [1 −1 0]. During the milling process, the cutting force was acquired by dynamometer. After cutting, in order to detect whether the micro-slots were in ductile removal mode or not, the slot surfaces were measured by an ultra-depth three-dimensional microscopy. The three-dimensional topography features of machined slot surfaces were measured by the white light interferometer.

**Figure 2:**
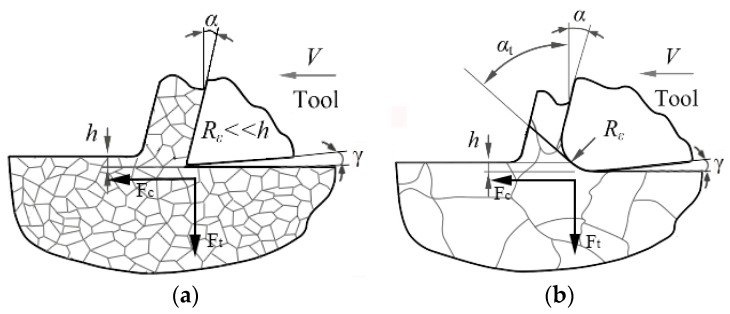
The effective rake angle during cutting process, F_c_ means feed force and F_t_ means thrust force. (**a**) Traditional machining manner; (**b**) micro-machining removal manner.

When processing single-crystal silicon with large diameter tools, feed force becomes dominant and the injury pattern is mainly caused by the formation of rake face wear. However, if small radius tools were used, the cutting force in thrust direction (thrust force) is paramount and the broad crater wear on angular flank face is serious, as shown in Figure 2. The reason is that the micro-processing mode always possesses very small uncut chip thickness and depth of cut in comparison to conventional machining operations. Once the uncut chip thickness is on the same level as the tool edge radius, the effective rake angle would become negative in Equations (1) and (2).

(1) αt=arcsin(h Re−1) for h<hc

(2) $$\alpha_{t}=a\;\;\mathrm{for}\;h>h_{c}$$

where *α_t_* is effective rake angle, *R_e_* is tool edge radius, *h* is uncut chip thickness, and *h_c_* can be calculated by *R_e_* (1 + sin*α*), which is the critical value. Actually, according to the plasticity theory, the magnitude of thrust force always determines the plastic deformation degree of fracture. Therefore, large negative rake tools were widely adopted to provide pressure stress field into the substrate surface during the ductile-model manufacturing process.

**Figure 3:**
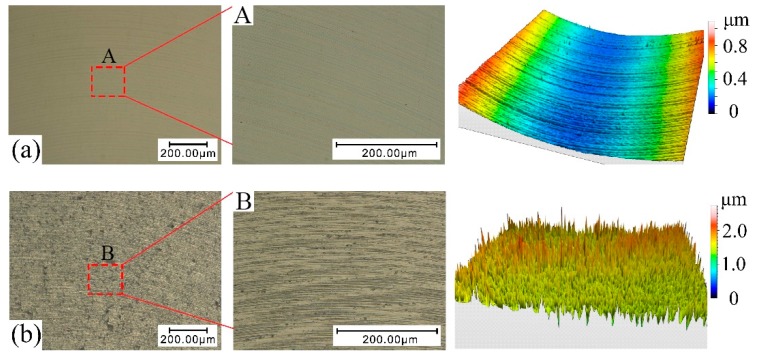
Selected machined surface topographies and three-dimensional morphology features at various cutting conditions. (**a**)—Test 2; (**b**)—Test 8.

**Figure 4:**
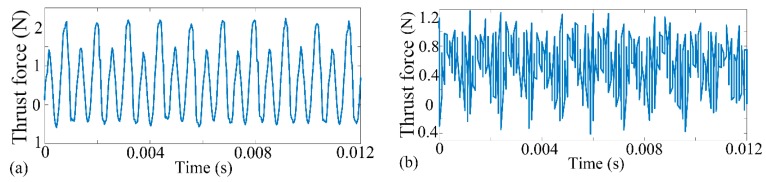
Selected measured cross force during micro-milling process. (**a**)—Test 2; (**b**)—Test 8.

From Table 1, it worth noting that the shape-structure and amplitude of thrust force kept close relations with the processing mode. Stable thrust force contributed to improving surface performance and inhibiting micro-defect initiation. However, once the boundary of stable cutting conditions was broken down, thrust force would become intermittent and disorganized. To further reveal the influence of thrust force state on micro-slot surface topography and processing precision, selected finished topography features and corresponding thrust force signals were shown in Figure 3 and Figure 4, respectively. In the present study, the morphology and distribution of surface defects were applied to determine the milling mode. From Figure 3a, we can see that the machined surface is smooth without any damages or fractures, which marks that the plastic removal mode has been achieved. Meanwhile, the three-dimensional morphology feature indicates that the machining paths are regular and clear. It is worth emphasizing that the cutting force profile is smooth and continuous without the vibration and halt as well, as shown in Figure 4a. Instead, plenty of micro-defects and micro-cracks are found in Figure 3b. Accordingly, the topography feature suggests that the surface characterized with brittleness processing is full of rugged mill-paths and chaotic burrs, which significantly affect the forming accuracy of the component. Accordingly, the signal of thrust force is fibrillated and intermittent in Figure 4b. 

**Figure 5:**
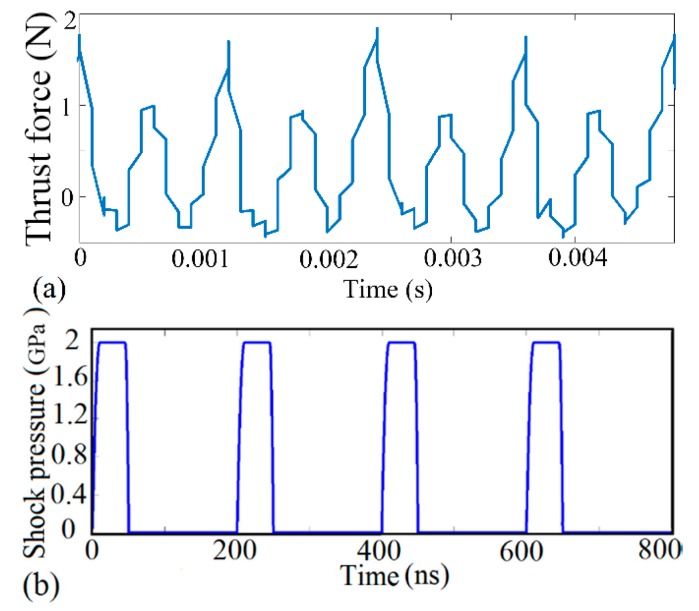
(**a**) Measured thrust force in experiment; (**b**) loading condition of shock wave in simulation.

According to the regulations above, the stable thrust force is helpful to inhibit surface defects and improve removal efficiency. However, chaotic thrust force could induce the generation of micro-cracks and micro-damages in finished surface. The reason may be that an unstable milling process always results in discontinuous impact effect, which produces approximate stress waves into monocrystalline silicon substrate [11], as shown in Figure 5a. Through adding impulse load into the multiscale simulation framework, this article attempts to dig into the formation mechanism of surface damage. Since the measured irregular cutting force signals usually demonstrate either transient singularity or continuous instability under partial ductile as well as brittle processing conditions, the dislocation evolution patterns of monocrystalline silicon were analyzed under the conditions of transient and continuous irregular cutting processes, respectively. Furthermore, as the thrust force mainly focuses on the effective contact region between the cutting edge and workpiece surface during the micro-milling process, the shock pressure on monocrystalline silicon at the cutting zone was calculated as the quantity ratio of cross force and contact cutting edge area. The stress wave evolved periodically with time, and the cycle time reduced to 200 ns to improve calculation efficiency. Meanwhile, the single cycle process was classified into four stages in Figure 5b: Increase stage of loadings (10 ns), remain stage of loadings (35 ns), decrease stage of loadings (5 ns), and no-load stage (150 ns). It is worth mentioning that the simulation model focuses on the effect of the incident wave and isolates reflected wave by adding the non-reflective boundary condition.

## 3. Multiscale Discrete Dislocation Plasticity Framework

**Figure 6:**
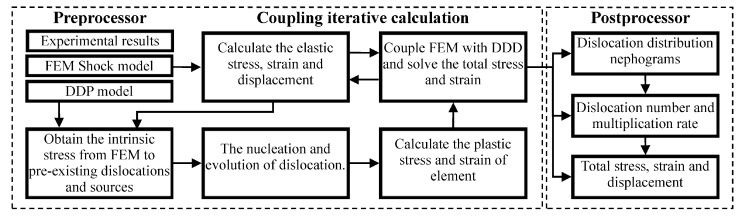
Multiscale discrete dislocation plasticity framework model.

A multiscale DDP model was employed to study the interaction relation between surface microstructure and irregular cutting process. This model incorporated two length scales, nano scale and continuum scale [30]. In the nano scale, discrete dislocation methods were adopted to determine the plasticity behavior of monocrystalline silicon by an explicit evolution mechanism of dislocations and interaction among themselves and other possible defects [31]. In the continuum scale, elastic driving force was developed on the basis of continuum mechanics laws [32]. This led to a hybrid elasto-viscoplastic multiscale simulation framework coupling dislocations into finite element method, as shown in Figure 6. 

In this framework, displacement, strain, and stress fields can be calculated by superposing singular field (^~^) and smooth image field (^) [33,34].

(3) $$u=\tilde{u}+\hat{u}\;\varepsilon =\tilde{\varepsilon }+\hat{\varepsilon }\;\sigma =\tilde{\sigma }+\hat{\sigma }$$

where the singular field (^~^) is attributed to the individual dislocations:

(4) $$\tilde{u}=\sum_{k=1}^{N}{\tilde{u}}^{k}\;\tilde{\sigma }=\sum_{k=1}^{N}{\tilde{\sigma }}^{k}$$

where *N* is the number of individual dislocations and {${\tilde{u}}^{k}$, ${\tilde{\sigma }}^{k}$} are the displacement and stress fields induced by *k*^th^ dislocation. The Peach–Koehler (*P-K*) force, which controls the evolution of dislocations, is shown:

(5) $$f_{g}^{\left(i\right)}=m^{i}\left({\hat{\sigma }}_{i}+\sum_{k\neq i}{\tilde{\sigma }}_{i}^{k}\right)b^{i}$$

(6) $$f_{c}^{\left(i\right)}=-s^{i}\left({\hat{\sigma }}_{i}+\sum_{k\neq i}{\tilde{\sigma }}_{i}^{k}\right)b^{i}$$

where *f_g_*^(*i*)^ is dislocation slip force, *f_c_*^(*i*)^ is dislocation climb force, *b*^(*i*)^ is the Burger’s vector, *m*^(*i*)^ and *s*^(*i*)^ are the unit vectors [35]. The slip velocity of dislocation *i* in-plane can be calculated by:

(7) $$v_{g}^{\left(i\right)}=M_{g}f_{g}^{eff(i)}$$

where *M_g_* = 1/*B_g_* and *B_g_* is friction coefficient. Friction force *f_g_^eff^* is introduced by considering the influence of Peierls–Nabarro (*P-N*) model. Frank–Read (*F-R*) sources with specific nucleation strength are randomly arranged in crystal slip planes. To avoid all dislocation sources being activated at the same time, the strength of each dislocation source must comply with Gaussian distribution. Moreover, *F-R* source can generate a dipole of edge dislocations if the *P-K* force exceeds the intrinsic nucleation strength in enough time:

(8) $$t_{F-R}=\frac{\eta }{2}\frac{Bl}{\tau_{F-R}b}F\left(\xi \right)$$

where *η* is 1.5 and *F*(*ξ*) is a decaying function. The distance between multiplication dislocation dipoles is in accord with following equation:

(9) $$L_{F-R}=\frac{G}{2\pi \left(1-v\right)}\frac{b}{\tau_{F-R}}$$

where *G* is Shear modulus, *v* is Poisson’s ratio. 

In this work, three-dimensional dislocation features, namely junction and lock, were both incorporated into two-dimensional plane simulations to enable the dynamic evolution of dislocation sources and obstacles [36]. Also, nano-scale precipitates and forest dislocations would form powerful obstacles on slip planes. Specifically, pinned dislocations can break above bondages if they are endowed with specific external stress. Moreover, the independent dislocations with equal and opposite Burgers vectors may be annihilated if their distance is less than a cut-off distance of 6*b*.

**Figure 7:**
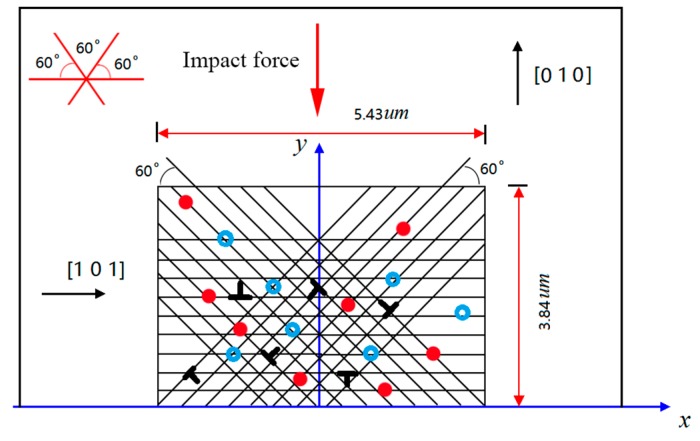
The periodical monocrystalline silicon model. The mesoscale material model consists of dislocation sources (solid circles), obstacle sources (open circles), pre-existing dislocations, and slip system.

By construction, a 5.43 μm × 3.84 μm representative cell (RC) was modelled to perform the DDP simulation during the plastic-cutting process, as shown in Figure 7. Following a previous study, the dislocation of silicon crystal glides on {111} slip system [37]. Therefore, the arbitrary set of slip system met plane stain restrictions and corresponding slip directions were {0°, 60°, 120°}. Considering thermal shock effect in the micro-manufacturing process, this model exerted a declining temperature distribution top-down. Selected impact force was applied into the simulation domain from the −*y* direction. The influence of boundary conditions on the elastic modulus and Poisson’s ratio was taken into account in the constitutive equation to better reveal the physical essence. In addition, *F-R* sources density is 5 × 10^12^ m^−2^, obstacle source density is 2 × 10^12^ m^−2^, Burgers vector is 0.384 nm, and viscosity coefficient is 2.6 × 10^−2^ 1/(Pa·s).

## 4. Results and Discussion

### 4.1. Dislocation Evolution in Silicon during Transient Irregular Cutting Process

**Figure 8:**
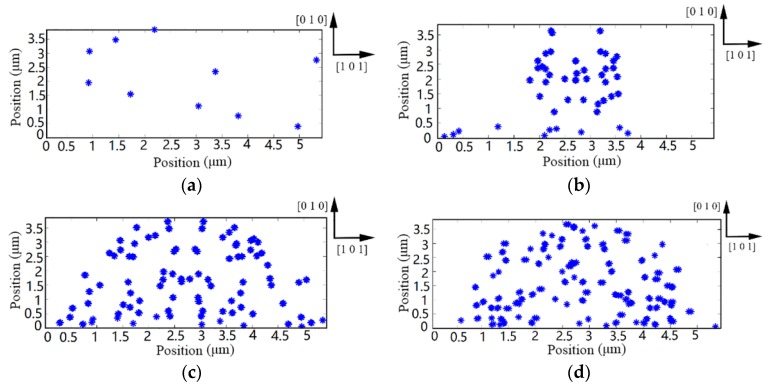
Dislocation patterns of monocrystalline silicon during transient irregular cutting process. (**a**) Dislocation structure at 5 ns; (**b**) dislocation structure at 15 ns; (**c**) dislocation structure at 30 ns; (**d**) dislocation structure at 50 ns.

Snapshots of dislocations–shock wave interaction were obtained to reveal the multiplication and propagation mechanism of dislocations during the transient irregular cutting process. Figure 8 shows the local evolution of dislocations microstructure during the transient irregular cutting process. A shock deformation study with pre-existing dislocations is confined into the calculation domain, as shown in Figure 8a. During the transient cutting process, dislocations are in continuous dynamic interaction with shock wave until the crest passes through dislocation configuration. In this simulation, once the extreme pressure surpasses the theoretical strength threshold of multiplication, a mass of dislocations would be generated from *F-R* sources. Upon propagating stress waves into the monocrystalline silicon surface, dislocations arrange themselves into certain morphologies. From Figure 8b, dislocation nucleation first appears in the directly beneath of substrate. Then, the multiplication region expands to both sides of the representative cell as the spreading of the stress wave, as shown in Figure 8c. At the same time, dislocation obstacles form local barriers to impede dislocation movement. In particular, due to the low self-diffusion of monocrystalline silicon, restricted dislocation cannot bypass the dislocation obstacles and impurity particles by climbing [38]. Therefore, most movable objects are obstructed at the end of loading stages, as shown in Figure 8d. It is worth noting that the dislocation configurations are consistent with the microstructure arrangement of monocrystalline silicon after warm laser shock peening [39].

**Figure 9:**
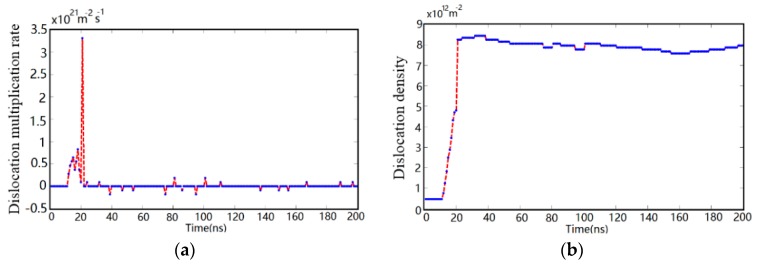
The evolution process of dislocation multiplication rate and total dislocation density during transient irregular cutting process. The red line represents the significant change of variables, and the blue line represents the gradual evolution of variables. (**a**) Dislocation multiplication rate evolution; (**b**) dislocation density evolution.

The evolution process of dislocation multiplication rate and total dislocation density against time in the transient irregular cutting process is presented in Figure 9. In the transient cutting process, dislocation nucleation plays a leading role in plastic deformation. From Figure 9, dislocation multiplication starts from 12 ns and the multiplication rate increases exponential with the rise of the value of cutting load. The dislocation density increases from 0.479 × 10^12^ m^−2^ to 8.441 × 10^12^ m^−2^ with the dislocation density evolution rate increased from 2.878 × 10^20^ m^−2^ s^−1^ to 3.453 × 10^21^ m^−2^ s^−1^. The transient energy transferred by shock wave results in that the pre-existing dislocations of silicon device make it difficult to produce a timely response for the energy impulse, which leads to the mushrooming of dislocation density and multiplication rate instantaneously, as shown in Figure 9a. In Figure 9b, the plateau region of the curve marks that dense dislocation configurations are the main performance of monocrystalline silicon under transient impact load. In addition, the curve of dislocation density evolution subsequently fluctuates within a narrow range due to dislocation annihilation and local nucleation. The predications are consistent with previous research, which indicates that the physics has been well captured [20].

### 4.2. Dislocation Evolution in Silicon during Continuous Irregular Cutting Process

Although tremendous cutting force would change the dislocation friction coefficient of silicon crystal, its free-evolution velocity is slow and inefficient in the no-load stage. To accelerate the simulation process, an appropriate promotion of dislocation activity under no-load condition was conducted. Note that the above adjustment would not affect the simulation results.

**Figure 10:**
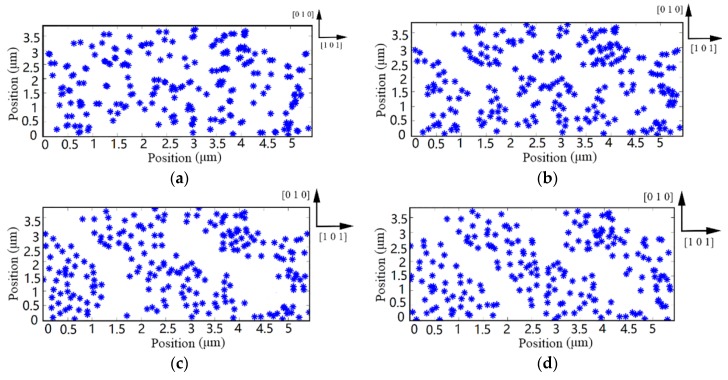
Dislocation patterns of monocrystalline silicon during continuous irregular cutting process. (**a**) Dislocation at 200 ns; (**b**) dislocation at 800 ns; (**c**) dislocation at 1400 ns; (**d**) dislocation at 2000 ns.

Figure 10 shows the microstructure evolution under the continuous irregular cutting process. The evolution of dislocation is a kinetic process, which is determined by the duration of pile-up at obstacles. With the changing of the dislocation pattern, a mass of dislocations may encounter the disturbance of local obstacles, such as forest dislocation, stacking fault tetrahedral, defect cluster, dislocation junction, and precipitate. These defects interact with dislocations within a short range and affect their configuration, as shown in Figure 10a,b. Although poor self-diffusion seriously hinders climbing, the continuous irregular milling process could provide enough energy to release pinning dislocations from bondages. Therefore, we can see that the quantity of immobile dislocations and forest dislocations significantly decreases when the simulation time is more than 1400 ns. Initially, a stable structural distribution is found in Figure 10a. Then, the vertical cross force frequently breaks original substrate microstructure so that the dislocation pattern is disconnected and disjointed, as shown in Figure 10b. However, although the dislocation patterns are repetitively sheared, broken dislocation configurations recover structural integrity all the time. Finally, the choroid structure gradually shortens and coarsens in Figure 10c,d. The above phenomenon marks that the substrate surface of monocrystalline silicon has formed persistent slip bands (PSBs) structure. 

**Figure 11:**
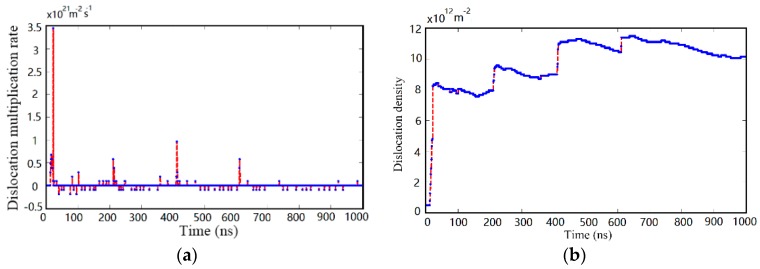
The evolution process of dislocation multiplication rate and dislocation density during continuous irregular cutting process. The red line represents the significant change of variables, and the blue line represents the gradual evolution of variables. (**a**) Dislocation multiplication rate evolution; (**b**) dislocation density evolution.

Since the wide fluctuation of dislocation density most converges in the first 1000 ns, Figure 11 concentrates on the changing of dislocation multiplication rate and dislocation density in this range. The poor locomotivity of dislocation in silicon crystal impedes energy release by slipping so that the substrate surface has to generate a mass of dislocation dipoles to preserve the plastic deformation. The dislocation multiplication mainly derives from the interaction between internal stress and shock loading so that the point-in-time of dislocation generation always appears at the turning point between the shock load stage and no-load stage, as shown in Figure 11b. Furthermore, it is worth noting that the amplitude of the dislocation multiplication rate curve presents slight increases so frequently, which can be explained by that the substrate takes place in local secondary nucleation to relieve residual stresses during previous several cycles. Following the growth of dislocation population, the quantity of residual dislocation sources decreases sharply. Meanwhile, the fluctuation of dislocation multiplication rate and dislocation density then weakens, which indicates that the total amount of dislocations has turned to be relatively stable. The phenomenon directly promotes the generation of PSBs in the later phase because the monocrystalline silicon substrate has to adjust its microstructures to accommodate external load. This result also accords with the microscale dislocation configurations discussed in the above results, as shown in Figure 10.

### 4.3. Mechanical Property and Damage Mechanism of Monocrystalline Silicon

**Figure 12:**
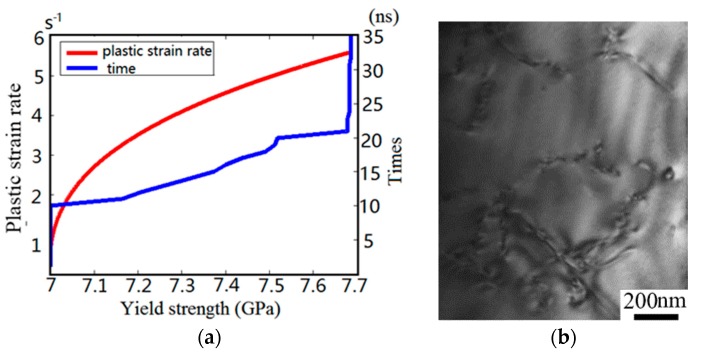
(**a**) The evolution of yield strength during transient irregular cutting process; (**b**) the TEM images obtained from transient loaded silicon crystal [39].

Plastic strain is attributed to the interaction between dislocations and boundary conditions. The plastic strain rate of monocrystalline silicon substrate can be calculated in light of the mended Orowan equation:

(10) $${\dot{\gamma }}_{p}=\phi b\rho \frac{d\bar{l}}{dt}+\phi b\bar{l}\frac{d\rho }{dt}$$

where *ϕ* is set as 0.408 according to Ref. [40], *ρ* is the density of mobile dislocation, *b* is Burgers vector and $\bar{l}$ is the average distance traveled by dislocation. In order to reveal the evolution of surface strength under transient impact process, the curves of yield strength against time and plastic strain rate are shown in Figure 12a.

**Figure 13:**
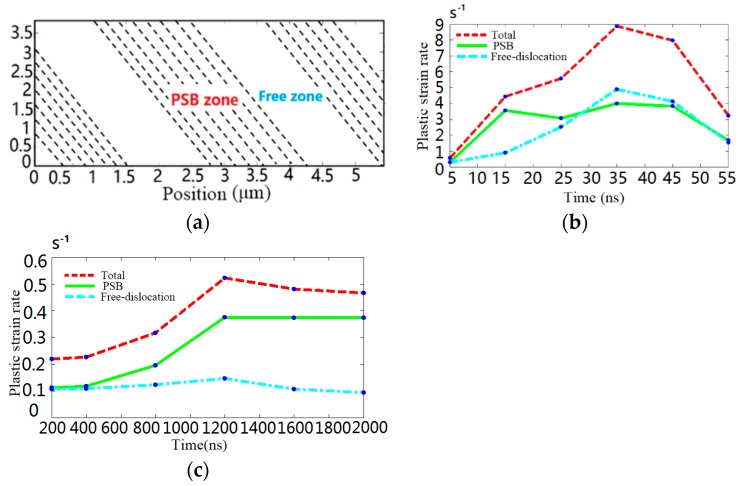
The plastic strain rate evolution of monocrystalline silicon. (**a**) The regional division of monocrystalline silicon; (**b**) monocrystalline silicon strain rate during under transient irregular cutting process; (**c**) monocrystalline silicon strain rate during continuous irregular cutting process.

Dislocation activity decides crystal deformability. Figure 12a indicates that a short-term shock wave can significantly improve the yield strength of silicon. The transient irregular cutting process generates compressive micro-internal stress in the surface and subsurface of silicon devices, which results in a dislocation entanglement structure and causes a dislocation self-interaction effect. The tangled structure not only inhibits the subsequent motion of dislocation on identical slip plane but also leads to machined surface work-hardening. Corresponding to this, as demonstrated in Cheng’s experiments [39], the plastic deformation can be generated in monocrystalline silicon by a laser shock peening experiments, which showed that shock pressure could lead to the tangled structure among dislocations and improve the ability of anti-deform in Figure 12b. Furthermore, in order to reveal the failure essence of brittle material, the RC model has been divided into the PSBs zone and free-dislocations zone in Figure 13a. Meanwhile, the evolution law of plastic strain rate under transient shock wave and continuous shock wave are shown in Figure 13b,c, respectively.

**Figure 14:**
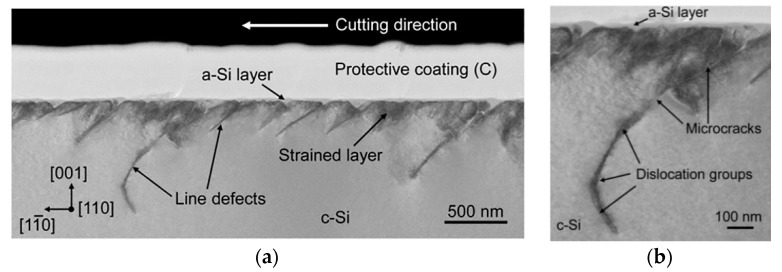
XTEM micrographs of a sample cut along 0° direction: (**a**) general view; (**b**) closeup bright and dark field views [41].

Since the velocity of mobile dislocation is proportional to the *P-K* force vector, the curve of Figure 13b has higher amplitude. As shown in Figure 13b, the dislocation velocity sharply increases with the increment of imposed load, yet then it drops significantly due to the obstacle particles and forest dislocations. By comparison, the plastic strain rates and dislocation structure in the PSBs zone and free-dislocations zone are nearly uniformity in early stage. However, Figure 13c demonstrates that the plastic strain rates in various zones exert a clear differentiation within the continuous cutting process. As PSBs structure possesses a higher dislocation density and free-dislocation structure keeps a lower dislocation density, the crystal plasticity deformation mostly concentrates upon the PSBs zone in a short time. This inhomogeneous deformation results in serious stress and strain concentration within monocrystalline silicon grains. In particular, the squash and stretch effect between the PSBs structure and free-dislocation structure may induce the initiation of micro-crack. The results agree well with the previous experiments, as shown in Figure 14 [41]. It indicates that numerous dislocation defects and PSB structures were formed in the machined subsurface during micro-cutting monocrystalline process.

## 5. Conclusions

Both experimental and simulation research work have been conducted to understand the surface damage formation process during the ductile-mode micro milling of single crystal Silicon. The main conclusions are listed as following:

1. Experimental results show that the morphology and amplitude of cutting force are closely connected with machined surface quality and surface roughness during the micro-machining monocrystalline silicon process. Stable thrust force can enhance ductile mode milling. On the contrary, the vibration thrust force often results in the brittle model removal.
2. The novel multiscale discrete dislocation dynamics modeling technique can reveal the interaction mechanism among dislocations and other defects. The predication for the evolution of dislocation structure falls within the existing experimental ranges, which indicates that the discrete dislocation simulation model developed in this study is robust and stable to study the plastic behavior of monocrystalline silicon in mesoscale.
3. The dislocation multiplication rate of monocrystalline silicon increases rapidly with the rise of impact loading. In particular, transient impact loading can form dislocations entanglement structure to improve surface yield strength of monocrystalline silicon.
4. The continuous irregular cutting process can induce persistent slip bands (PSBs) structure, which causes the inhomogeneous deformation and stress concentration within grains.

## Figures and Tables

**Table 1:** The machining conditions for the milling operations and responses: measured surface finish, cutting force mode, and milling mode.

Test No.	Axial Depth of Cut (μm)	Feed Rate (μm/Tooth)	Spindle Speed (rpm)	Surface Roughness ^1^ Ra (nm)	Cutting Force Mode ^2^ (S/PS/C)	Milling Mode ^3^ (D/PD/B)
1	10	0.075	50,000	9.292	S	D
2	30	0.075	50,000	12.835	S	D
3	60	0.075	50,000	13.352	S	D
4	120	0.075	50,000	127.724	C	B
5	30	0.3	50,000	19.671	S	D
6	30	0.6	50,000	15.984	PS	PD
7	60	0.3	50,000	89.999	PS	PD
8	60	0.6	50,000	108.877	C	B

^1^ Surface roughness values presented in the above table are the mathematic average for measured Ra. ^2^ S is smooth mode; PS is partial smooth mode; C is chaos mode. ^3^ D is ductile mode; PD is partial ductile mode; B is brittle mode.
